# YAP at the progression of inflammation

**DOI:** 10.3389/fcell.2023.1204033

**Published:** 2023-06-15

**Authors:** Libin Chen, Xintong Jin, Jian Ma, Bo Xiang, Xiayu Li

**Affiliations:** ^1^ Department of Gastroenterology, The Third Xiangya Hospital of Central South University, Changsha, China; ^2^ Hunan Key Laboratory of Nonresolving Inflammation and Cancer, The Third Xiangya Hospital of Central South University, Changsha, China; ^3^ The Key Laboratory of Carcinogenesis and Cancer Invasion of the Chinese Ministry of Education, Cancer Research Institute and School of Basic Medical Sciences, Central South University, Changsha, China

**Keywords:** inflammation, YAP, macrophages, NF-κB, IFN-I, inflammatory disease

## Abstract

Yes-associated protein (YAP) is a transcriptional regulator that affects cell proliferation, organ size and tissue development and regeneration, and has therefore, been an important object of study. In recent years, there has been an increasing research focus on YAP in inflammation and immunology, and the role of YAP in the development of inflammation and in immune escape by tumors has been progressively elucidated. Because YAP signaling involves a variety of different signal transduction cascades, the full range of functions in diverse cells and microenvironments remains incompletely understood. In this article, we discuss the complex involvement of YAP in inflammation, the molecular mechanisms through which it exercises pro- and anti-inflammatory effects under different conditions, and the progress achieved in elucidating the functions of YAP in inflammatory diseases. A thorough understanding of YAP signaling in inflammation will provide a foundation for its use as a therapeutic target in inflammatory diseases.

## 1 Introduction

Yes-associated protein (YAP) is a downstream effector in the Hippo pathway. It typically functions as a transcriptional coactivator dependent on other transcription factors. Early research on YAP established that it is an initiator of carcinogenesis that promotes tumor growth and invasive migration (Huang et al., 2005). Increasing evidence suggests that YAP plays a critical role in the regulation of innate and adaptive immunity. According to recent research, YAP functions in the progression of inflammatory diseases including inflammatory bowel disease, atherosclerosis, osteoarthritis, and pneumonia (Nowell et al., 2016; Yang et al., 2019; Zhou et al., 2019). Understanding the mechanisms of interaction between YAP and inflammatory factors, the roles of YAP in inflammatory pathways, and the regulatory functions of YAP in the development of inflammatory illnesses will provide critical foundational knowledge for the development of new therapeutic strategies. This review discusses the known pro- and anti-inflammatory actions of YAP in various cell types and microenvironments, and mechanisms of action in regulating inflammatory illnesses.

## 2 Function and regulation of YAP involving inflammation

**FIGURE 1:**
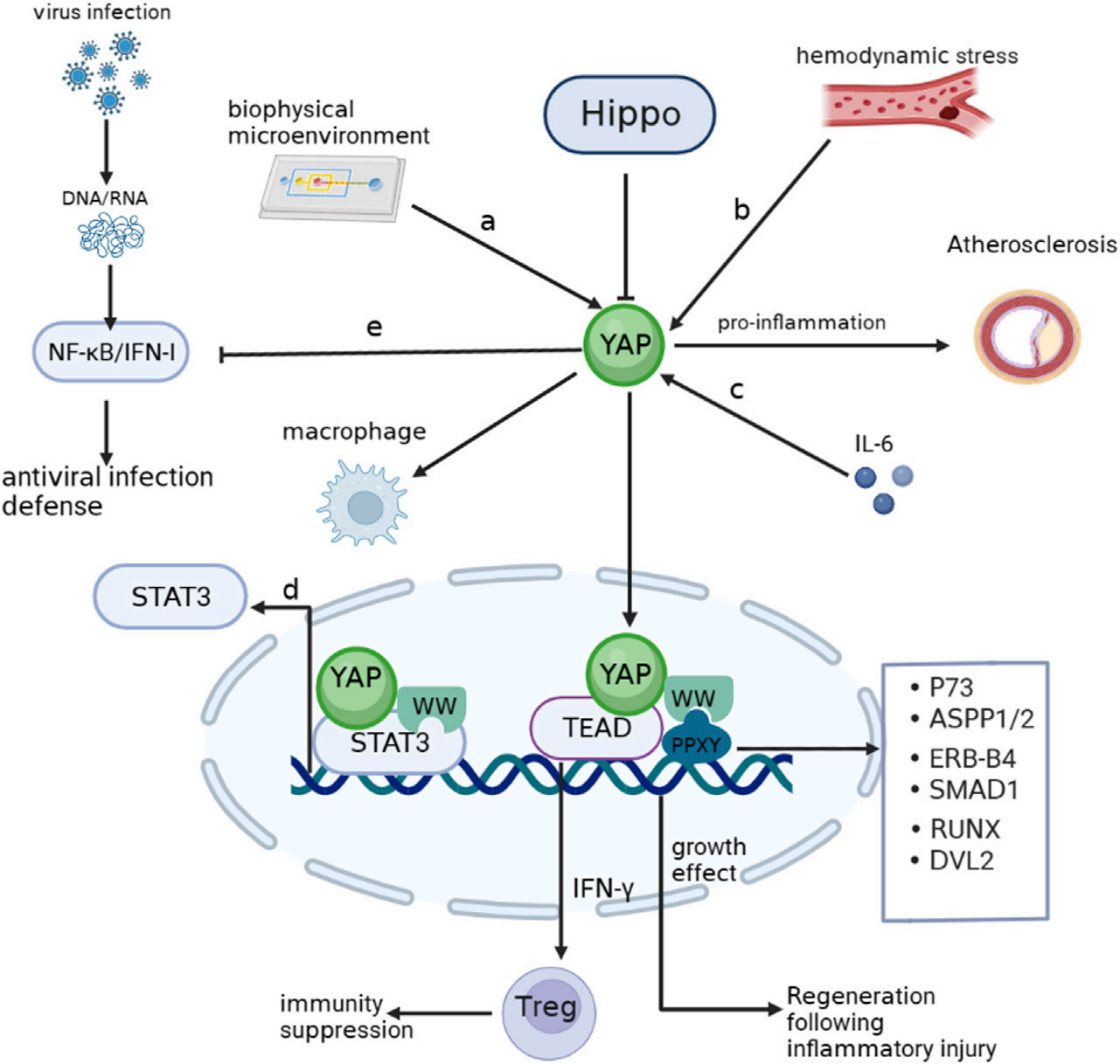
YAP involving inflammation **(A)**. YAP enters the nucleus when cells adhere to hard surfaces and enhances the macrophage response to inflammation. **(B)**. Hemodynamic alterations activate YAP to promote atherogenesis. **(C)**. IL-6 activates YAP to promote inflammatory repair aftr injury. **(D)**. The WW domain of YAP can bind to the STAT3 promoter and stimulate transcription. **(E)** NF-κB/IFN-Ⅰ signaling activated by viral infection is inhibited by YAP. Arrows indicate activation, blunt ends indicate inhibition. P73, tumor protein p73; ASPP1/2, protein phosphatase 1 regulatory subunit 13B; ERB-B4,erb-b2 receptor tyrosine kinase 4; SMAD1, SMAD family member 1; RUNX1, RUNX family transcription factor 1; DVL2, disheveled segment polarity protein 2.

YAP plays a crucial role as a sensor of cell structure, which encompasses important biological properties such as cell polarity, cytoskeletal shape, and the impact of alterations in the adhesion microenvironment on YAP expression (Dupont et al., 2011; Fernández et al., 2011; Zhao et al., 2012). Previous studies indicate that the cell adhesion microenvironment regulates the inflammatory response of macrophages by influencing the intracellular localization of YAP. Adherence of macrophages to soft hydrogels, instead of hard materials, results in a reduction in YAP expression and nuclear localization and the secretion of inflammatory factors (Meli et al., 2020). In contrast, active YAP in macrophages enhances inflammatory activation. This observation aligns with a recent study conducted on Kupffer and THP-1 cells, where binding of YAP to promoters of inflammatory genes through TEAD motifs (a member of the TEA structural domain family) was observed (Zhou et al., 2019; Song et al., 2020). Furthermore, altered hemodynamics in response to oscillatory shear stress or TNF-α stimulation enable YAP to form a complex with BTB domain and CNC homolog 1 (BACH1), promoting the expression of inflammatory genes and adhesion molecules in atherosclerotic plaques (Jia et al., 2022). Notably, following myocardial infarction, YAP recruits Treg cells by increasing IFN-γ level and counteracts the post-infarction inflammatory response (Ramjee et al., 2017). Furthermore, the inhibitory impact of YAP on NF-κB/IFN-I signaling, which is triggered by the infected host in response to viral invasion, will be elaborated in subsequent sections as we delve into the precise underlying mechanism. YAP contains a WW structural domain that is vital for its transcriptional co-activation function. There is an interaction between the WW domain of YAP and the PPXY motif on P73 to promote a specific response to DNA damage (Strano et al., 2005). Signal transducer and activator of transcription 3 (STAT3) serve as a link between inflammation and cellular transformation. YAP can be recruited by JUNB, a member of the activator protein (AP)-1 family, as well as by STAT3, stimulating the transcriptional activation of AP-1 and STAT3 through direct interaction between the WW structural domain and STAT3 and JUNB (He et al., 2021). Other PPXY-containing proteins, such as Angiomotin-like, ASPP1/2, ERB-B4, SMAD1, RUNX, and DVL2, interact with the WW structural domain within the Hippo-YAP pathway (Salah and Aqeilan, 2011). Importantly, Studies have revealed that IL-6 triggers the self-renewal of cultured embryonic stem (ES) cells by activating YAP (Taniguchi et al., 2015). This finding indicates that inflammation-activated YAP serves as a mechanism to counteract inflammation in addition to contributing to cell growth. These findings imply that YAP remains primarily dedicated to its original function, further emphasizing its role in regulating cellular responses to inflammation (Figure 1).

## 3 YAP pro-inflammatory effects

**FIGURE 2:**
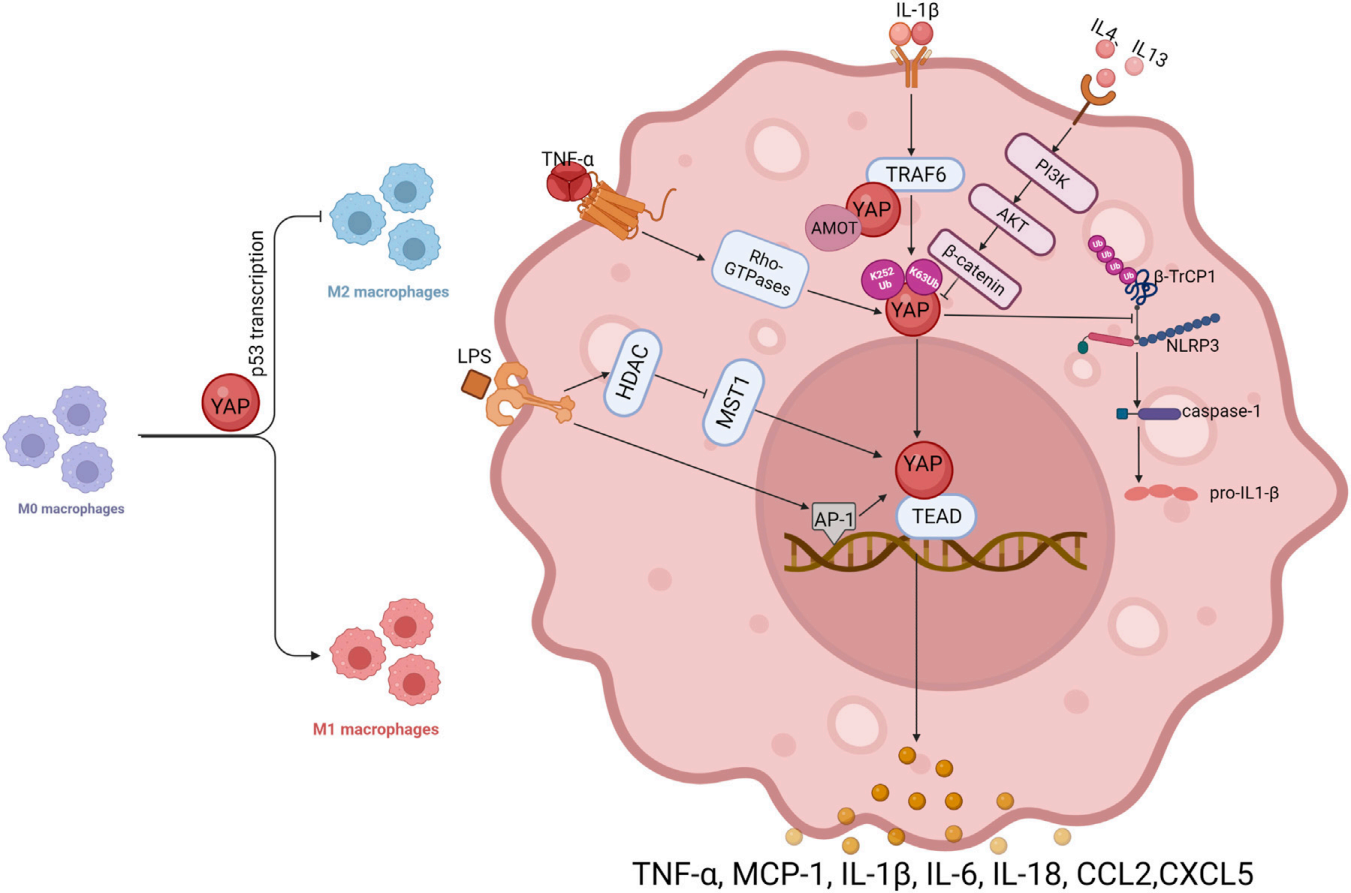
In macrophages, YAP interacts with inflammatory factors.

Macrophages, as the main mediators of inflammation, have been used to study the role of YAP in inflammation. Macrophages acquire different functional phenotypes depending on the microenvironment. Two well-recognized polarization phenotypes involve classically activated (M1) and alternately activated (M2) macrophages (Mosser and Edwards, 2008). High expression of YAP promotes macrophage polarization toward the M1 type, whereby YAP inhibits M2 polarization in macrophages by promoting transcription of p53 (Zhou et al., 2019). The data suggest that macrophages respond to inflammatory stimuli depending on nuclear levels of YAP, with exclusion of YAP from the nucleus preventing inflammation (Meli et al., 2020). (Figure 2)

Most studies to date have shown that YAP has a pro-inflammatory function in macrophages, and several inflammatory factors can promote YAP transcriptional activity in macrophages, forming a positive feedback loop in the inflammatory response. Tumor necrosis factor (TNF)-α can promote the expression of YAP, and can also activate YAP through the Rho family of GTPases, promoting nuclear translocation of YAP and stimulate inflammatory gene expression (Caire et al., 2022). IL-1β treatment increases YAP protein levels in macrophages in a dose- and time-dependent manner, but mRNA levels do not change significantly, implying that IL-1β induces an increase in YAP protein levels in macrophages by a post-translational mechanism. It has been suggested that IL-1β increases YAP accumulation by inhibiting proteasomal degradation. In macrophages, IL-1β enhances YAP stability by activating tumor necrosis factor receptor associated factor 6 (TRAF6), promoting interaction between YAP and TRAF6, stabilizing YAP protein by promoting TRAF6-induced K63 YAP ubiquitination and reducing K48 YAP ubiquitination. In addition, TRAF6-mediated YAP ubiquitination on K252 interrupts binding to AMOT, eliminates AMOT-mediated cytoplasmic retention of YAP, promotes nuclear translocation, and increases secretion of chemokines including chemokine ligand 2 (CCL2) and CXCL5 by macrophages (Liu et al., 2020). YAP can also prevent ubiquitination and degradation of the nucleotide-binding and leucine-rich repeat (NLR) family pyrin domain-containing 3 (NLRP3) inflammasome. It is widely recognized that an unregulated NLRP3 inflammasome drives pathogenesis in inflammatory diseases (Davis et al., 2011). In murine macrophages, As a result of YAP’s interaction with NLRP3, NLRP3 is maintained in its stable state by preventing the association between NLRP3 and the E3 ligase *β*-TrCP1, which promotes NLRP3 degradation via K27-linked ubiquitination at lys380. Stable NLRP3 can activate caspase-1 to promote expression of IL-1β and exacerbate inflammation (Wang et al., 2021). In addition, telomere dysfunction can drive ATM-mediated YAP activation, which directly promotes transcription of key inflammatory corpuscle genes and upregulates the expression of pro-IL-18. In response to the colon microbiome, caspase-1 is activated, which cleaves pro-IL-18 into mature IL-18 (Chakravarti et al., 2020), which stimulates the recruitment of interferon (IFN)-γ-secreting T cells and intestinal inflammation.

Endotoxic lipopolysaccharide (LPS) of intestinal microbial origin activates macrophage differentiation to the M1 pro-inflammatory phenotype via Toll-like receptor 4 (TLR4) (Miura and Ohnishi, 2014). According to prior studies, LPS/TLR4 triggers the nuclear factor κB (NF-κB), activator protein (AP)-1, and interferon regulatory factor 3 (IRF3) cascades leading to pro-inflammatory effects (Abu-Shanab and Quigley, 2010; Machado and Cortez-Pinto, 2012). It is necessary for NF-κB activation and TNFα production in macrophages to be stimulated by LPS through YAP activation and nuclear translocation, As well, the interaction between YAP and NF-κB subunit p65 provides a structural basis for the nuclear localization of p65 and the production of pro-inflammatory cytokines (Yang et al., 2020a). AP-1 is a critical transcription factor in the inflammatory response (Trop-Steinberg and Azar, 2017). In response to LPS treatment, the AP-1 motif in the YAP promoter is more tightly bound, leading to increased transcription of YAP. LPS signaling can also activate YAP through histone deacetylase (HDAC)-mediated deacetylation of MST1, resulting in degradation of MST1 by lysosomes (Li et al., 2016; Song et al., 2020). In addition, YAP protein stimulates the expression of pro-inflammatory cytokines (such as MCP-1, TNF-α, and IL-6) by associating with the TEAD-binding motif in the promoter region of inflammatory cytokine genes.

The anti-inflammatory factors IL-4 and IL-13 can inhibit YAP expression in macrophages and reduce the production of inflammatory factors through the PI3K-AKT-β-catenin pathway, which further demonstrates the pro-inflammatory effect exerted by YAP in macrophages. The evidence reveals that YAP exacerbates progression of inflammation in inflammatory diseases, especially in macrophages, where YAP interacts with inflammatory factors to generate positive feedback in the inflammatory response and promote development of inflammatory cytokine storm. At the same time, another role of YAP, in tissue repair and anti-inflammatory aspects of the inflammatory response, has gradually emerged.

Activation of YAP promotes P53 transcription, inhibits macrophage polarization from M0 to M2, and promotes M0 differentiation to M1; in macrophages, TNF-α activates YAP through Rho-GTPase, and IL-1β ubiquitinates YAP at K63 through TRAF6 to stabilize YAP; TRAF6 also directly interacts with YAP to keep AMOT away from YAP and restore free and nuclear localization of YAP. LPS activates AP-1, promotes YAP transcription, and activates YAP through HDAC-mediated degradation of MST1; YAP prevents the ubiquitination and degradation of NLRP3 by *β*-TrCP1, maintains NLRP3 stability, and then activates caspase-1, which catalyzes the synthesis of IL-1β; the anti-inflammatory factor IL-4/IL-13 can inhibit the expression of YAP in macrophages through PI3K-AKT-β-catenin. Arrows indicate activation, blunt ends indicate inhibition, Ub indicates ubiquitylation site. PI3K, phosphatidylinositol 3-kinase; AKT, AKT serine/threonine kinase.

## 4 YAP anti-inflammatory effects

**FIGURE 3:**
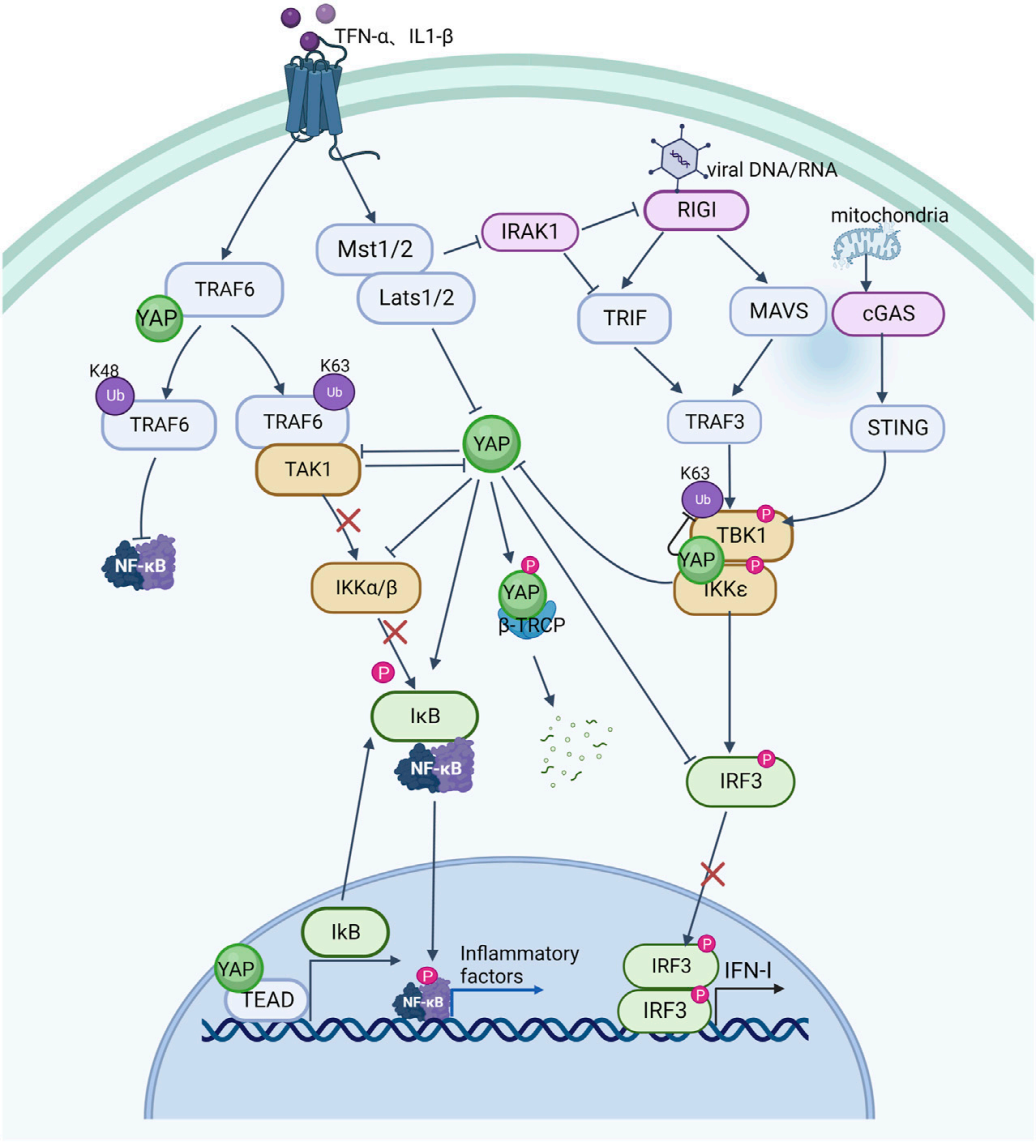
The crosstalk between YAP pathway and NF-κB signaling and innate antiviral response.

Emerging evidence suggests that the YAP signal cascade regulates inflammatory signals, and that activation of innate immune signals inhibits YAP signal activation. Several studies have reported that YAP signal deficiency is associated with immunodeficiency symptoms, including in bacterial and viral infection, implying that YAP also seems to play an important role in suppressing the progression of inflammation (Figure 3).

### 4.1 YAP and NF-κB signaling

Nuclear factor kappa B (NF-κB) signaling is critical in inflammation and immune responses, and various signals activate NF-kB to promote transcription of inflammatory factors by activating IkappaB protein (IκB) kinase (IKK) to degrade IκB. TNF-α and IL-1β have been shown to promote K63 ubiquitination of TNF receptor associated factor 6 (TRAF6) in endothelial cells to further activate NF-κB signaling (Lawrence, 2009). YAP can bind to TRAF6 and promote K48 ubiquitination of TRAF6 to induce TRAF6 degradation while inhibiting K63 TRAF6 ubiquitination, thereby inhibiting NF-κB activation and preventing excessive inflammatory responses to endotoxin (Lv et al., 2018). Other studies have also demonstrated that YAP can interact with TGF-beta activated kinase 1 (TAK1) and reduce IKK phosphorylation of IκBα by reducing TAK1 substrate accessibility to recruit IKK and directly inhibiting IKKα/β activation. When YAP is overexpressed, ubiquitination of IκBα is greatly reduced and the interaction between IκBα and *β*-TRCP is weakened, further inhibiting IκBα degradation as well as p65 activation and nuclear translocation, leading to diminished NF-κB signaling activity (Deng Y. et al., 2018). YAP can also bind to the promoter of IkB together with the transcription factor TEAD to promote transcription of IkB and prevent NF-κB from translocating to the nucleus (LaCanna et al., 2019). TNF-α and IL-1β can directly activate phosphorylation of YAP via the LATS1/2 kinase of the Hippo pathway, resulting in SCF/β-TRCP-E3 ubiquitin-ligase-mediated cytoplasmic retention and degradation of YAP, which can also occur by means of TAK1-mediated YAP phosphorylation. *β*-TRCP-mediated ubiquitination promotes proteasomal degradation of YAP (Deng F. et al., 2018). These findings indicate a reciprocal inhibitory relationship between YAP and NF-κB signaling, whereby increased YAP expression in inflammation may limit NF-κB activation and control the inflammatory response.

### 4.2 YAP and IFN-I signaling

A host’s defense against viral infection is characterized by the production of type I interferon (IFN-I, including IFN-α and IFN-β). Multiple interferon-stimulated genes (ISGs) are produced by IFN-I, which protects host cells from virus invasion. YAP has been found to be involved in antiviral processes and is a negative regulator of innate antiviral responses that can inhibit the IFN-β signaling pathway (Wang et al., 2017). Typically, exogenous and endogenous nucleic acids activate the IKK pathway in response to viral infection as well as cellular damage leading to mitochondrial DNA exposure. The IKK kinase is activated by DNA and RNA sensors, such as RNA sensor (RIG-I), interferon induced with helicase C domain 1 (MDA-5), mitochondrial antiviral signaling protein (MAVS), cyclic GMP-AMP synthase (cGAS), and stimulator of interferon response cGAMP interactor 1 (STING1), leading to the production of IFN-α and IFN-β, and activates the body’s protective mechanisms. As along with pro-inflammatory cytokines, and activating the body’s protective responses (Kawai and Akira, 2010; Takeuchi and Akira, 2010). YAP interacts with interferon regulatory factor 3 (IRF3), the final downstream effector of IKK signaling and a key transcription factor in innate immunity, preventing dimerization and nuclear translocation, which reduces IFN-β production in response to viral infection. This inhibition of IRF3 by YAP is mainly mediated by YAP4, a YAP isoform without an amino terminal, unable to interact with the TEAD domain, which suppresses the antiviral response of IRF3 more efficiently. Viral infection, on the other hand, can cause YAP degradation via activation of IKK. IKK can phosphorylates YAP at S403 and induces its degradation by lysosomes. Whereas depletion of IKK reduced phosphorylation and degradation of YAP S403 upon infection with a virus. Due to YAP’s significant inhibitory effect on innate antiviral immunity, as part of the body‘s defense against viral infection, phosphorylated YAP is degraded by IKK dependent lysosomal enzymes (Wang et al., 2017). Consistent with this, MST1, an upstream inhibitory kinase acting on YAP, phosphorylates IL-1 receptor-associated kinase 1 (IRAK1), this results in the degradation of IRAK1 (Li et al., 2015). IRAK1 is a negative regulator of TRIF- and RIG-I-mediated IFN-β signaling (An et al., 2008; Bruni et al., 2013). IRF3 is therefore activated and IFN-β is produced in response to the degradation of IRAK1 is mediated by MST1. In addition, YAP has also been shown to suppress innate antiviral defenses by targeting TBK1. YAP prevents TBK1 from being ubiquitinated at K63 and inhibits TANK binding kinase 1 (TBK1) interaction with MAVS, STING, and IRF3, consequently, IFN-I increases caused by viruses are reduced (Zhang et al., 2017). Recently, ATP6V0d2 (a V-ATPase subunit) was found to promote lysosomal degradation of YAP. Viral infection or serine metabolism defects increase expression of ATP6V0d2 by inhibiting S-adenosylmethionine-dependent H3K27me3 occupancy at the promoter, alleviating YAP-mediated blockade of the TBK1-IRF3 axis and thereby increasing IFN-β production (Shen et al., 2021). Mice experiments have demonstrated that innate antiviral responses are enhanced and virus susceptibility is reduced by genetically deleting YAP (Wang et al., 2017).

In summary, YAP suppresses innate antiviral immune responses, whereas activation of innate antiviral immunity can lead to YAP degradation and protect host cells from viral damage. YAP signaling can act as an inflammatory signaling switch to prevent excessive inflammation and organ failure.

TNF-α and IL-1β activate K63-linked ubiquitination of TRAF6, promote the interaction between TRAF6 and TAK1, activate IKKα/β kinase, and then phosphorylate and degrade IκB, relieve the inhibition of NF-κB signaling by IκB, allow it to enter the nucleus from the cytoplasm, and promote the transcription and expression of inflammatory factors; YAP can bind to TRAF6, inhibit K63-linked ubiquitination of TRAF6, activate K48-linked ubiquitination of TRAF6, cause TRAF6 degradation and inhibit NF-κB signaling; YAP also prevents TAK1 from recruiting IKK and directly inhibiting the activation of IKKα/β to inhibit NF-κB signaling as well as stimulate IκB transcriptional expression and prevent NF-κB from entering the nucleus. However, TNF-α and IL-1β can activate MST1/2 and LATS1/2, so that YAP is retained and destroyed in the cytoplasm through SCF/-TRCP E3 ubiquitin ligase; TNF-α and IL-1β can also increase NF-κB signaling through TAK1-mediated YAP phosphorylation and degradation. Viral DNA and RNA can activate RIG I. RIG I interacts with MAVS and TRIF to stimulate TRAF3, which then promotes TBK1 and IKK interaction and their phosphorylation, dimerizes IRF3 and nuclear translocation, and increases the expression of IFN-I. In addition, exposure of mitochondrial DNA can also activate TBK1 and increase the expression of IFN-I through cGAS and STING; YAP can directly hinder IRF3 dimerization and nuclear translocation, as well as inhibit TBK1 K63 ubiquitination, and block its interaction with MAVS, STING and IRF3, reducing the expression of IFN-I; but IKK activation in response to viral infection can also cause the degradation of YAP; and MST1-mediated IRAK1 (negative regulatory molecule of RIG I and TRIF) degradation increases the activation of IRF3 and the production of IFN-I. Arrows indicate activation, blunt ends indicate inhibition, Ub indicates ubiquitylation site, P indicates phosphorylation, The red x indicates an interrupted signal path.

## 5 YAP involvement in inflammatory disease

**FIGURE 4:**
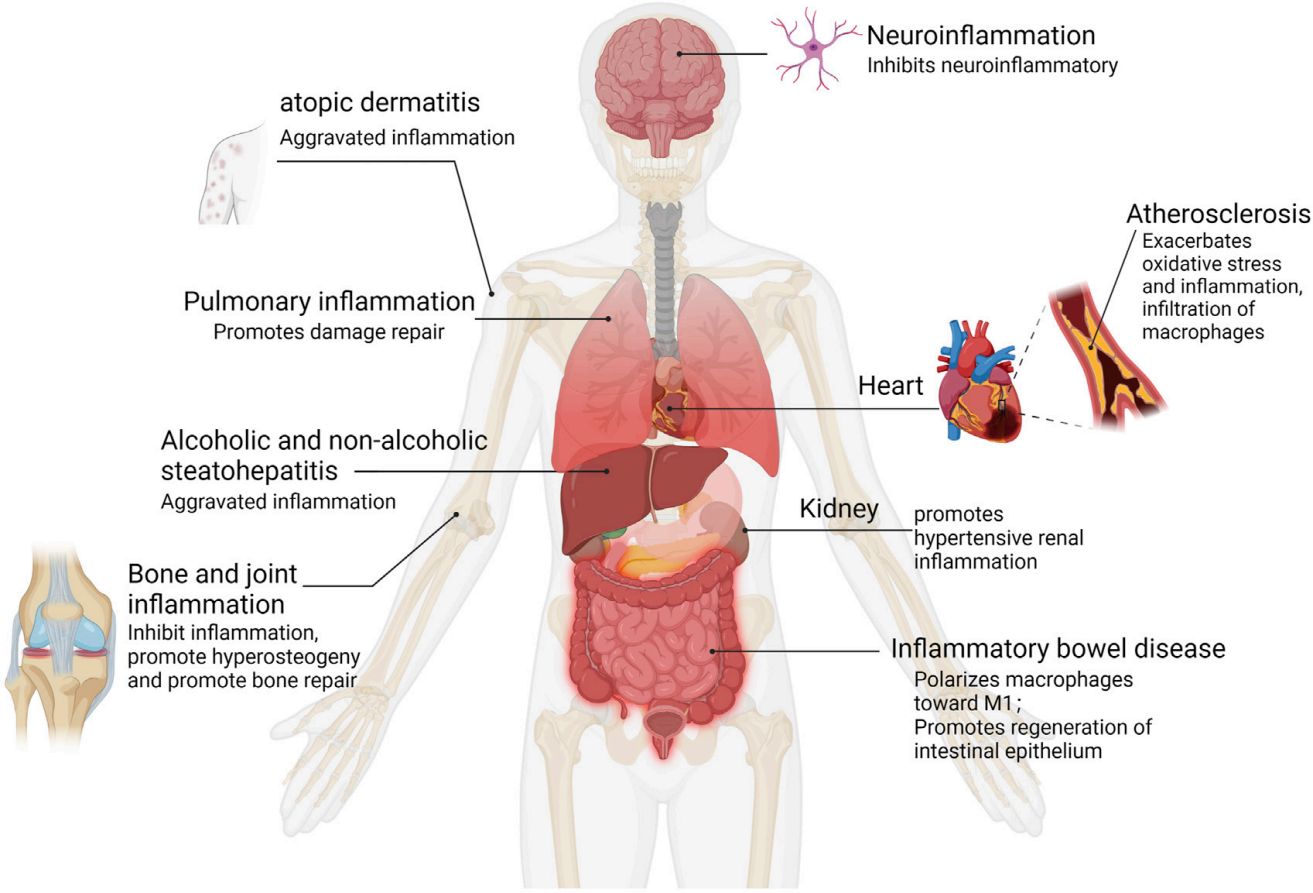
YAP activation in human inflammation. Inflammation types for which epidemiological data and functional evidence of YAP activation have been reported.

The outline of the interaction of YAP with inflammatory pathways and inflammatory factors summarizes general themes. In the following sections, we discuss what is known about YAP in human inflammatory diseases and what has been learned from experimental models (See Figure 4 and Table 1).

**TABLE 1:** YAP expression and functional relevance in human inflammation and experimental models.

Inflammation types	Specimens	Factors	Description contents	Citation
acute lung injury	Mouse model	TRAF6 NF-κB	YAP modulates the activation of endothelial cells and Reduce inflammatory damage to lungs through preventing TRAF6-mediated NF-κB activation	(Lv et al., 2018)
lung inflammation	Mouse model	AECII IκBa NF-κB	In bacterial pneumonia, AECII needs YAP to activate IκBa in order to prevent NF-κB-mediated inflammation and encourage alveolar epithelial recovery	(LaCanna et al., 2019)
inflammatory lung injury	Cell lines Mouse model	mtDNA	mtDNA-cGAS signaling induces YAP phosphorylation, preventing YAP-mediated endothelial proliferation and repair and exacerbating lung injury	(Huang et al., 2020)
Atherosclerosis	Tissue Cell lines Mouse model	IL-1β TRAF6 Macrophage	TRAF6 induces increased nuclear localization of YAP in macrophages, accelerating atherosclerosis	(Liu et al., 2020)
Atherogenesis	Cell lines Mouse model	disturbed flow	YAP is activated by disturbed flow to promote the phenotype of atherosclerosis	(Wang et al., 2016a)
Atherogenesis	Mouse model	BACH1	BACH1 increases YAP expression, which promotes atherogenesis and vascular inflammation	(Jia et al., 2022)
Atherogenesis	Tissue Cell lines Mouse model	integrin-Gα13 JNK	Integrin-G1α3 inhibits YAP and JNK signaling, delaying atherogenesis	(Wang et al., 2016b)
Inflammatory Bowel Disease	Mouse model	IL6 Macrophage	YAP can exacerbate IBD by promoting IL6 production and M1 macrophage polarization	(Zhou et al., 2019)
Intestinal Inflammation	Mouse model	miR-590-5p	MiR-590-5p relieves intestinal inflammation by inhibiting YAP	(Yu et al., 2018)
Intestinal Inflammation	Tissue Cell lines Mouse model	intestinal epithelial cell (IEC)	YAP-driven IEC proliferation can promote inflammatory recovery	(Deng et al., 2018a)
Intestinal Inflammation	Tissue Cell lines Mouse model	PGE 2	PGE 2-targeted YAP encourages colon tissue regeneration	(Kim et al., 2017)
Osteoarthritic	Tissue Cell lines Mouse	NF-κB	YAP suppresses NF-κB signaling and is involved in OA cartilage healing	(Deng et al., 2018b)
Osteoarthritic	Cell lines Mouse model	MiR-582-3p	MiR-582-3p alleviates osteoarthritis progression by targeting YAP1	(He et al., 2020)
neuroinflammation	Cell lines	Sirt3	When YAP is downregulated, Sirt3 is inhibited and JNK is activated, which may lead to neuroinflammation	(Yang et al., 2019)
autoimmune encephalomyelitis	Mouse model	Astrocytic	YAP also promotes proliferation of astrocytes and induces cholesterol synthesis genes (e.g., HMGCS1), preventing neuroinflammatory infiltration	(Zhang et al., 2021a)
alcoholic hepatitis	Tissue Mouse model	hepatocyte transdifferentiation	Aberrant activation of YAP plays an important role in hepatocyte transdifferentiation in AH, through a loss of hepatocyte identity and impaired regeneration	(Bou Saleh et al., 2021)
alcoholic hepatitis	Mouse model	CXCL1	YAP activation leads to increased expression of CXCL1, causing neutrophil infiltration and liver inflammation	(Kusumanchi et al., 2021)
Nonalcoholic steatohepatitis	Tissue Mouse model	Kupffer Cells	YAP increases in Kupffer Cells and levels of YAP positively correlate with expression of pro-inflammatory cytokines	(Song et al., 2020)
hypertensive nephropathy	Mouse model	Angiotensin (Ang) II	Angiotensin (Ang) II activates YAP and promotes hypertensive renal inflammation and fibrosis	(Zhang et al., 2021b)
Atopic dermatitis	Cell lines	LEKTI HaCaT cells	YAP overexpression downregulated lympho-epithelial Kazal type inhibitor (LEKTI) levels and aggravated viability and inflammation in TNF-α and IFN-γ-treated HaCaT cells	(Cheng et al., 2020)

### 5.1 Pulmonary inflammation

YAP plays a protective role in lung inflammation and injury repair. Selective knockout of YAP in mouse lung epithelium can lead to lung cysts similar to those of emphysema (Lin et al., 2017). YAP expression in alveolar endothelial cells can prevent ventilator-induced lung injury, and the absence of YAP in endothelial cells can cause inflammation and activate NF-кB signal transduction in animal models of acute lung injury, leading to increased pulmonary vascular permeability and inflammation (Lv et al., 2018). YAP can regulate NF-κB Activity and promote IκBa transcription by targeting IκBa in type II alveolar epithelial cells (AECII). The recovery of IκBa expression in YAP mutant lung promotes regression of inflammation. Deletion of YAP in AECII reduces alveolar epithelial regeneration mediated by surfactant protein C and leads to prolongation of fibrosis in the lung during bacterial pneumonia (LaCanna et al., 2019). Recent studies have shown that, in an experimental model of inflammatory lung injury, mtDNA activates cyclic GMP-AMP synthase (cGAS) signaling and inhibits YAP-mediated endothelial cell proliferation, such that the regeneration ability of endothelial cells is impaired, promoting inflammatory injury (Huang et al., 2020). These observations suggest that YAP may promote repair following inflammatory injury in the lung.

### 5.2 Alcoholic and non-alcoholic steatohepatitis

Chronic alcohol consumption is a major risk factor for alcoholic hepatitis (AH), and uncontrolled YAP activation has been found to be a key factor in the pathogenesis of AH. The whole liver was immunohistologically examined and RNA-seq analysed in AH samples and microdissected hepatocytes showed abnormal activation of YAP in hepatocytes. Persistent activation of YAP in AH hepatocytes deprives them of hepato-cyte characteristics while differentiating towards the cholangiocyte program. In mouse models, When YAP is overactivated in hepatocytes *in vitro* and *in vivo*, biliary differ-entiation occurs and critical functions such as regeneration are lost. In contrast, thera-peutic inhibition of YAP activity blocked transdifferentiation of hepatocytes from AH patients and transform abnormal hepatocytes into mature hepatocytes (Bou Saleh et al., 2021). In addition, the upstream kinase of yes associated protein (YAP), MST1 was inhibited in ethanol-fed mice, affecting their ability to phosphorylate YAP and phosphorylation of YAP in hepatic cells is inhibited by ethanol, which resulted in YAP nuclear translocation and TEAD1 activation. CXCL1 is increased in expression following activation of TEAD1, a chemokine-mediated recruitment of neutrophils causing neutrophil infiltration and liver inflammation (Kusumanchi et al., 2021).

Nonalcoholic steatohepatitis (NASH) is an inflammatory disorder in which exces-sive fat accumulates in the liver without excessive alcohol consumption. It has been found that YAP expression is significantly increased in Kupffer cells (KCs) from wild-type mice fed a high-fat diet (HFD), and analysis of liver tissue from NASH pa-tients shows that YAP is increased in KCs, and a positive correlation exists between YAP levels and pro-inflammatory cytokines in human liver tissue, and Kupffer cells (KCs) produce pro-inflammatory cytokines when YAP is activated and promotes the development of nonalcoholic steatohepatitis (NASH) (Song et al., 2020). Other studies have also confirmed that chronic inflammation inhibits activation of Hippo kinase, leads to increased activity of downstream YAP, and promotes hepato-biliary carcinogenesis resulting from chronic damage to liver inflammation (Hyun et al., 2021).

### 5.3 Atherosclerosis

Atherosclerosis is characterized by chronic inflammation of the arterial wall, associated with endothelial dysfunction and structural changes. Studies have shown that YAP activation occurs in vascular endothelial cell (EC) inflammation induced by oxidized low-density lipoprotein (ox-LDL), which promotes oxidative stress and inflammation (Yang Y. et al., 2020). Overexpression of YAP in bone marrow cells increases the size of atherosclerotic lesions and infiltration by macrophages (Liu et al., 2020), and blood laminar flow is disturbed during plaque formation, activating YAP and increasing nuclear translocation (Wang K. C. et al., 2016). Activation of YAP increased JNK inflammatory signal transduction, and BACH1 combined with the YAP promoter to upregulate YAP expression, inducing expression of adhesion molecules and inflammation in endothelial cells, promoting the progression of atherosclerosis (Wang L. et al., 2016; Jia et al., 2022). YAP knockdown significantly attenuated EC proliferation and pro-inflammatory factor expression; inhibition of YAP activation may thus represent a promising strategy for atherosclerotic protective treatment. Naringin reduces ox-LDL-triggered apoptosis of human umbilical vein endothelial cells and expression of the inflammatory factors IL-1, IL-6, and IL-18 by inhibiting the YAP pathway (Wang K. C. et al., 2016). Recombinant IL-1 receptor antagonists reduce the ubiquitination of YAP triggered by TRAF6 at K252, thereby promoting interaction between YAP and angiomotin, inhibiting nuclear translocation of YAP, and reducing the formation of atherosclerotic lesions (Liu et al., 2020).

### 5.4 Inflammatory bowel disease

YAP has been widely studied in inflammatory bowel disease (IBD). Recent findings (Zhou et al., 2019) showed that IBD is aggravated by YAP by affecting macrophage polarization in M1/M2 macrophages. YAP can also aggravate IBD by combining with IL-6 promoters to promote M1 macrophage polarization and IL-6 production. YAP knockout in DSS-induced colitis mice promoted M2 macrophage polarization, and significantly increased the mRNA levels of the anti-inflammatory cytokines Arg1, Fizz, and IL-10. Other studies have shown that YAP expression is upregulated significantly in intestinal epithelium of IBD patients and in mice with colitis induced by TNBS. Other investigators have also found similar results that inhibition of YAP expression is mediated by Mir-590-5p targeting the 3′-untranslated region of YAP gene, which alleviates colitis in mice (Yu et al., 2018). In epithelial regeneration in a DSS-induced mouse colitis model, high YAP expression enhanced the self-renewal of mouse epithelial cells, restored the structure of the intestinal recess, and improved the wound healing ability of IEC (Deng Y. et al., 2018). Some studies have shown that prostaglandin E2 (PGE-2) signaling increases the expression and transcriptional activity of YAP1, resulting in the increased expression of cyclooxygenase-2 and prostaglandin E2 receptor 4, activating a positive feedback loop and promoting regeneration of colonic epithelial cells in colitis mice (Kim et al., 2017). Overall, existing studies have shown that YAP can participate in immune regulation and promote the progress of inflammation in immune macrophages in the stage of intestinal inflammation, while in the stage of inflammation recovery, YAP activation in intestinal epithelial cells promotes recovery of intestinal epithelial barrier function.

### 5.5 Bone and joint inflammation

YAP can inhibit bone and joint inflammation and promote bone hyperplasia and repair. In TNF-α-induced inflammation, YAP overexpression inhibits the expression of IL-6 and NF-κB in TNF-α-treated osteoclasts. YAP inhibits TNF-α-induced activation of the NF-κB signaling pathway by phosphorylating p65, promoting p65 nuclear translocation and inhibiting expression of the NF-κB gene, thereby promoting proliferation and differentiation of osteoblasts (Yang B. et al., 2020). YAP is necessary for maintaining cartilage homeostasis in osteoarthritis. YAP can also regulate cartilage formation and differentiation of ATDC5 cells promoted by TNF-α through the AMPK signaling pathway (Chen et al., 2020). Other studies have also demonstrated that YAP inhibited the NF-κB-mediated inflammatory response in osteoarthritis mouse model. Cytokines secreted by the inflammatory response were inhibited by overexpression of YAP, including TNF-α, IL-1, and IL-6. Experimental mice overexpressing YAP or deleting the MST1/2 gene alleviated osteoarthritis symptoms, while deletion of YAP in chondrocytes aggravated osteoarthritis. Other work found that MiR-582-3p could inhibit chondrocyte apoptosis, reduce the production of pro-inflammatory cytokines, inhibit degradation of the extracellular matrix (ECM), and alleviate the progression of osteoarthritis by targeting YAP (He et al., 2020).

### 5.6 Neuroinflammation

Astrocytes play an important role in ion balance in the central nervous system and the normal development of the nervous system. It has been found that YAP is highly expressed in astrocytes. YAP-knockout astrocytes show excessive activation of the Janus kinase (JAK)-STAT inflammatory pathway and reactive astrogliosis, revealing that YAP negatively regulates neuroinflammation (Huang et al., 2016). Molecular studies have revealed that neuroinflammation can be driven by YAP downregulation, causing inhibition of Sirt3 and activation of JNK. However, YAP overexpression was able to protect BV-2 neuronal cells from TNFα-mediated apoptosis by regulating the Sirt3-JNK signaling pathway (Yang et al., 2019). Furthermore, YAP was found to be upregulated and activated in spinal astrocytes in experimental autoimmune cerebrospinal mice. YAP deletion in astrocytes aggravates experimental autoimmune encephalomyelitis (EAE), causing earlier onset, more severe inflammatory infiltration, a greater degree of demyelination, and neuronal loss. YAP prevents demyelination in EAE by promoting the expression of cholesterol synthesis genes (e.g., HMGCS1). Astrocyte YAP can prevent neuroinflammatory infiltration and demyelination by upregulating TGF-β signaling in optic neuritis (Zhang et al., 2021a; Wu et al., 2021).

## 6 Conclusion

According to current research, the effect of YAP on the inflammatory process is complex. YAP expression can accelerate the regression of inflammation in the bone joint, cartilage, and lung; however, it plays a role in the progression of atherosclerosis and has various functions at different stages of IBD. Additionally, YAP influences vascular endothelial cells and chondrocytes by inhibiting the NF-κB pathway, reducing inflammation, and accelerating recovery from inflammation and can also promote the transcription of inflammatory factors in macrophages, playing a pro-inflammatory role. Studies of rodent models of hepatectomy have demonstrated the importance of the transient activation peak of YAP during liver regeneration (Moya and Halder, 2019), and YAP protein was later shown to promote the progression of the early hepatocyte cycle (Tschuor et al., 2019). This finding contrasts with current data which unraveled that YAP activation prevents hepatocyte repair. We believe the important distinction should be made between transient and sustained activation of YAP. The cascade regulation and impact of external microenvironment signals on YAP in inflammatory processes need to be considered for a more comprehensive investigation. Overall, several key questions remain to be addressed in future studies; given that YAP expression modulation through knockdown or overexpression could potentially alter the onset, progression, and outcome of inflammation, a detailed understanding of NF-κB, STAT3, or other inflammatory pathways related to YAP is crucial. Additionally, investigating the impact of environmental rigidity on YAP and macrophages or other immune cells, such as NK cells, T cells, or neutrophils, is important. Furthermore, exploring the mechanisms underlying the mutual inhibition of YAP with NF-κB, IFN-I, or other factors involved in antiviral infection is necessary. A YAP-related signaling pathway could be proposed as part of the inflammatory cascade to develop new anti-inflammatory or anti-viral drugs that affect the upstream kinase activity of YAP, including MST1/2 and LATS1/2, which would be beneficial for the treatment of cancer and inflammatory diseases. Therefore, conducting clinical studies to explore the use of YAP signaling as a therapeutic target holds promise.
